# A veterinary virapalooza: a summary of the 2024 American Society for Virology (ASV) Veterinary/Zoonotic Virology Satellite Symposium and online H5N1 panel discussion

**DOI:** 10.1128/jvi.00499-25

**Published:** 2025-05-13

**Authors:** Andrew Broadbent

**Affiliations:** 1 Department of Animal and Avian Sciences, University of Maryland 1068 , College Park, Maryland, USA; Universiteit Gent, Merelbeke, Belgium

**Keywords:** veterinary virology, zoonoses, virology, agricultural viruses, H5N1

## Abstract

The year 2024 saw veterinary/zoonotic virology take center stage once more as the American Society for Virology (ASV) hosted a satellite symposium on the subject in June and an online panel discussion in December. The symposium comprised six talks from experts on viruses of economic importance to agriculture and of public health importance. The viruses in question spanned foot and mouth disease virus (FMDV), African swine fever virus (ASFV), Marek’s disease virus (MDV), severe acute respiratory syndrome coronavirus 2 (SARS-CoV-2), and influenza A viruses (IAVs), and topics covered fundamental virology, applied virology, epidemiology, and surveillance. The goal was to emphasize that improving the control of animal viral diseases requires an integrated, holistic approach involving academia, government, and industry labs undertaking research on basic virology, vaccinology, epidemiology, and surveillance. Moreover, the symposium aimed to highlight career opportunities in the agricultural/veterinary sector for those with virology training. Six months following the symposium, the ASV held an online panel discussion on the ongoing H5N1 IAV situation in poultry, cattle, and people to provide more up-to-date information to its membership. A summary of the talks and discussions is presented here.

## INTRODUCTION

The American Society for Virology (ASV) is committed to representing and supporting its members who specialize in viral diseases of animals. To this end, the 2024 annual meeting held in June at the Greater Columbus Convention Center in Columbus, Ohio, and hosted by The Ohio State University featured veterinary and zoonotic virology in the form of workshops, plenary speakers, state-of-the-art talks, and a satellite symposium. The veterinary virology community selected a broad theme for the symposium to encompass researchers working on One Health, non-zoonotic animal viral diseases, and vaccines and requested speakers from the industry and government in addition to the academia. As a result, the 2024 symposium was titled “A Veterinary Virapalooza: Improving the Control of Viral Diseases of Animals,” and the goal was to highlight that improving the control of animal viral diseases requires an integrated, holistic approach involving academia, government, and industry labs undertaking research on basic virology, vaccinology, epidemiology, and surveillance. To this end, the symposium was structured into sessions on fundamental virology, applied virology, and epidemiology and surveillance, with the latter also being a One Health theme tackling zoonoses. Moreover, a key aim of the symposium was to demonstrate career opportunities for virologists in the agricultural/veterinary sector.

Since the last veterinary virology satellite symposium in 2021, major animal virus outbreaks have continued, including African swine fever virus (ASFV) in Asia, Russia, and Europe (1), severe acute respiratory syndrome coronavirus 2 (SARS-CoV-2) in wild animals in the USA (2), foot and mouth disease virus (FMDV) in Germany (3), and H5N1 influenza globally (4, 5). The symposium provided an opportunity to address these critical issues, followed 6 months later by an online panel discussion on H5N1. Here, a summary of the presentations and discussions from the events is presented.

## VETERINARY/ZOONOTIC VIROLOGY SATELLITE SYMPOSIUM—JUNE 2024

### Fundamental virology

Prof. Nicola Stonehouse (Professor, Faculty of Biological Sciences, University of Leeds, UK) kicked off the day with a talk on FMDV replication. FMDV is a member of the *Picornaviridae *family affecting cloven-hoofed ungulates. Although the US is free of the disease without vaccination, it remains a priority pathogen that is notifiable to USDA due to trade concerns (6). Despite its importance, little is known regarding how the virus replicates in cells compared to other picornaviruses. Prof. Stonehouse described how, *in vitro*, wild-type FMDV polymerase proteins formed higher-order complex structures resembling fibrils. Although they are yet to be found in infected cells, when mutations were made in the polymerase to disrupt these higher-order interactions, some reduced the replication of FMDV replicons, suggesting that the complexes may play a functional role in the viral reproductive cycle. Moreover, FMDV viral RNA can form secondary structures, such as hairpins and pseudoknots. Four pseudoknots have been described in the genome. This seemed to provide a competitive advantage during replication, and when all were removed, no infectious virus could be recovered (7). Recently, Prof. Stonehouse’s collaborators discovered a potential packaging signal within the pseudoknot region adjacent to the poly-C tract that was critical for the recovery of virus, as well as several other potential non-essential but beneficial packaging signals elsewhere in the genome (8). Finally, a region known as the S fragment, located within the 5′ untranslated region, forms a long hairpin, and its removal also abolished replication. Truncation of the S fragment reduced replication of mutant viruses; however, after blind passage in cells, an amino acid mutation developed in the polymerase in a region that made close contact with the RNA (I189L) that partially compensated for the loss of replication incurred by the S fragment truncation (9). These studies demonstrate the intricate relationship between the polymerase and RNA, and understanding such interactions could inform the development of antiviral strategies.

Next, Dr. Douglas Gladue (Vice President of Veterinary Pharmaceutical Development at Seek Labs) gave a talk on ASFV, a double-stranded (ds) DNA virus belonging to the *Asfarviridae* family that affects wild boar, warthogs, and domestic pigs, with transmission occurring directly between pig and boar populations or via a tick (10). Infection can be asymptomatic or can lead to a variety of clinical signs, including fever, skin erythema, inflammation of the conjunctiva, vomiting, diarrhea, increased respiratory and heart rates, and lack of coordination. Such morbidity can be severe, and mortality percentages can be over 90% in some pig herds (10). Historically, ASFV had remained mainly in Africa, with only sporadic outbreaks outside of Africa, for example, in Europe, the Caribbean, and South America that were eventually controlled by widespread culling and changes in management practices. However, in 2007, an outbreak in the Republic of Georgia led to subsequent spread to Russia and Poland, and by 2018, the outbreak had reached China. From there, it spread rapidly across Southeast Asia, and in 2022, it was declared a panzootic (1). Rationally designed live attenuated ASFV vaccines have since been developed (11) and licensed, for example, one where six multi-gene families (MGFs) were deleted (ASFV-G-ΔMGF) (12), and one where a predicted immunomodulatory gene (*I177L*) was deleted (ASFV-G-ΔI177L) (13, 14). ASFV-G-ΔI177L represents the first commercially available vaccine for ASFV made by NAVETCO, and ASFV-G-ΔMGF is the second, but the first to be made in a stable cell line by AVAC. Dr. Gladue’s team has also been using machine learning to classify ASFV strains into different biotypes by comparing every open reading frame of diverse strains to create a matrix (15). Using this approach, seven biotypes have been identified (16). Dr. Gladue is now at Seek Labs, where he is using CRISPR technology to target the ASFV genome to disrupt virus replication without affecting host gene expression. This technique has achieved 57% survival of ASFV-infected pigs compared to 0% survival with no treatment, and it is hoped this could be used in an outbreak setting. His work illustrates how fundamental virology informs the development of control strategies.

### Applied virology

Dr. Claudia Osorio [Associate Advisor for US Poultry Elanco Animal Health, Inc., and President of the American Association of Avian Pathologists (AAAP), 2024–2025] delivered a presentation on the critical role of applied virology in poultry production. The US poultry industry is a global leader, producing 9.17 billion broilers (chickens sold for meat) in 2022, totaling to $76.9bn in sales (17). Given the scale of the industry, effective disease prevention measures, such as vaccination, are essential to prevent production losses and ensure flock health. Dr. Osorio highlighted the use of vaccines in commercial poultry settings, where up to 20 commercial vaccines are administered, depending on the breed, age, and specific disease risk. In addition to commercial vaccines, autogenous vaccines are used to target farm-specific pathogens. She emphasized the various methods used to administer vaccines, including inoculation into the embryonated egg (*in ovo*), which allows for high automation, delivery to the mucosal surface by spray or drinking water, and administration by subcutaneous or intramuscular injection. One such success story from the industry was the development of vaccines against Marek’s disease virus (MDV), a herpesvirus responsible for tumors in chickens. Before the introduction of the vaccine, MDV caused mortality rates between 30 and 80% in affected flocks. Today, thanks to vaccination, the disease is no longer clinically in the US. Moreover, the herpesvirus of turkeys (HVT) is a useful vector for the delivery of antigens to protect chickens against other diseases and is becoming popular in vaccine schedules (18). One main take-home from Dr. Osorio’s talk was that there is a pressing need for scientists with virology and immunology expertise to help make the next generation of poultry vaccines. These efforts are crucial for maintaining the health and productivity of poultry flocks and for preventing economic losses within the industry. Dr. Osorio highlighted that many virologists already have positions within the poultry industry in a variety of roles. Furthermore, Dr. Osorio encouraged members of ASV to explore career opportunities in the poultry industry and to get involved with the AAAP to strengthen links between the two societies.

### Epidemiology and surveillance

Addressing the topic of One Health, Dr. Andrew Bowman (Professor, College of Veterinary Medicine, The Ohio State University) gave a talk on SARS-CoV-2 in white-tailed deer. In January–March 2021, Dr. Bowman’s team processed nasal swabs from 360 deer from nine urban metro parks in Ohio and found that 129 (35.8%) were positive for SARS-CoV-2. The virus was found at all sites, and male deer had higher rates than female. Whole-genome sequencing was performed, and the entire genome sequences of 14 strains were obtained from six sites, and three lineages were discovered. Moreover, the lab was able to isolate two of the virus samples using Vero cells (19). Following on from this study, 83/88 counties in Ohio were tested, and nearly 60% had positive deer, with 163/1522 (10.7%) animals being antigen-positive and 274/1164 (23.5%) seropositive. The lab identified six independent human-to-deer transmission events, indicating that deer are highly susceptible to infection from people, and meaning that SARS-CoV-2 is a reverse zoonosis, as well as a zoonosis. Viruses were sequenced during the delta wave, yet two clusters were found to be the alpha variant, suggesting months of persistence in the deer population, which is different from the situation in humans. Interestingly, despite this persistence, the rate of evolution was threefold higher in deer compared to people (20). These data demonstrate how viruses can evolve differently in animal populations compared to humans, emphasizing the importance of continued surveillance.

Addressing companion animals, Dr. Colin Parrish (Professor, College of Veterinary Medicine, Cornell University) discussed the epidemiology of H3N8 and H3N2 influenza A viruses (IAVs) in dogs and horses. The H3N8 IAV first emerged in horses in South America in the 1960s before spilling over into dogs in 1999. Canine H3N8 was first recognized in greyhounds in a training facility in Florida and initially spread with greyhound movement in the US to many regions in the South and Midwest but then quickly began to die out, with phylogenetic analyses revealing only sustained transmission in geographically constrained areas around Denver and Colorado Springs and in the North East USA, being maintained in dog shelters in New York City. The virus eventually became extinct in the dog population in 2016 (21) but continues to circulate in horses.

In contrast, the H3N2 IAV resulted from an avian virus transferring to dogs around 2004 and was first reported from South Korea and China (22). Canine H3N2 then formed a Chinese lineage and a South Korean lineage. In 2015, canine H3N2 was first transmitted to North America in rescued dogs from South Korea that were brought to the US. Initially, it was believed that this virus subsequently died out similar to the H3N8 viruses (23), but the virus re-emerged in Florida in 2021, and that virus spread to the Los Angeles area soon after where it likely infected over 50,000 dogs. The evidence points to this being a separate reintroduction into the US, likely due to the importation of large numbers of dogs during the coronavirus disease 2019 pandemic, when so-called “pandemic pups” were becoming popular because of social distancing at the time.

Phylogenetic and Bayesian analyses suggest that canine H3N2 viruses died out in South Korea around 2017 but became established in China, after which they appear to have seeded recent North American outbreaks, with two or three introductions in the past 3 years (22). Genomic epidemiology conducted by Dr. Parrish’s team confirms that within North America, the H3N2 virus spreads very rapidly among dogs in kennels and shelters in different regions but then dies out locally, likely once the animals have become infected and immune. Household dogs in North America appear not to be connected enough to maintain the virus in circulation, so sustaining the epidemic requires the movement of the virus to more distant dog populations with dense populations where the virus can spread (22). Moreover, the strain is evolving at a constant rate, consistent with other influenza viruses, and has not yet gained properties that would keep the virus in prolonged circulation among dogs (22).

Sticking with the topic of influenza, Dr. Erica Spackman (Distinguished Senior Research Scientist, Exotic & Emerging Avian Viral Diseases Research, US National Poultry Research Center) gave a talk on the H5N1 IAV situation. Typically, low-pathogenicity avian influenza viruses can spill over from ducks to poultry populations. If poultry become infected with H5 or H7 subtypes, the disease may become highly pathogenic avian influenza (HPAI) for reasons that are poorly understood. HPAI can cause 100% mortality in infected poultry flocks, which can be rapid and accompanied by clinical signs, such as lethargy, neurological signs, and hemorrhagic lesions in chickens in wattles, combs, legs, and internal organs (24). Since H5N1 was first detected in wild birds in the US at the end of 2021, many spillover events have occurred from wild waterfowl into poultry, causing significant losses to the poultry industry (at the time of writing, 168.26 million poultry in 1,674 flocks have been affected by H5N1 in the USA since the outbreak in poultry began on 8 February 2022 [25]). Then, in early 2024, a reassortant strain emerged in wild birds and transmitted directly to cattle in the US and has since spread (26) (at the time of writing, 996 dairy herds in 17 states have been affected by H5N1 in the USA since the outbreak in cattle began on 25 March 2024 [27]). While the wild bird H5N1 strains mostly belong to genotype D1.1, the vast majority of cattle have been affected by genotype B3.13. Now, there is a breadth of species infected; the geographic range is wider; and the load of virus is high in resident birds maintaining it locally, so the landscape has changed (at the time of writing, there have been three separate spillover events of H5N1 into cattle, one of genotype B3.13 and two of genotype D1.1 [28]). Moreover, the H5 virus has also transmitted from cattle to poultry, and now poultry continues to be infected from the bovine B3.13 genotype in addition to aquatic waterfowl D1.1 genotype (29). Ducks shed higher titers of the virus for a longer time compared to gallinaceous avian species. In one study, chickens shed virus for 2–3 days post-exposure (24), whereas in another study, ducks shed virus for the duration of the experiment (14 days) (30). Ducks also have a wide geographic distribution, which could increase the exposure of other species, enhancing the potential for spillover into a broader range of hosts. Currently, the focus of control efforts has been to preserve the US export of poultry and dairy products, but open questions remain. For example, do we eradicate, do we depopulate, or do we vaccinate? The goals can be different depending on the industry, and it is important to note that vaccines only work well when there is good biosecurity in place. Several H5 vaccines are licensed in the US: an inactivated vaccine based on a reverse-genetics virus with a backbone comprising lab-strain PR8, an RNA particle vaccine comprising a non-replicating alphavirus vector, two recombinant HVT vectored vaccines, and a fowlpox vectored vaccine, which is not produced in the US anymore (31). However, trade restrictions exist for vaccinated production animals, and vaccinating can be labor-intensive, particularly if given intramuscularly or subcutaneously. Valuable birds have been vaccinated; for example, 140 condors were vaccinated with an inactivated H5 vaccine, which is an example of a vaccine success (32). Whether we vaccinate poultry depends on several factors, including the cost benefit, and whether a test can be developed to ensure differentiation of infected from vaccinated animals (at the time of writing, an inactivated H5 vaccine has been conditionally approved by the USDA for use in chickens [33], but a decision to vaccinate US flocks has not yet been made).

## H5N1 ONLINE DISCUSSION 12/11/24

Six months after the symposium, ASV hosted an online discussion on the ongoing H5N1 outbreak. This was the first online content delivered for its membership. Dr. Andrew Bowman (Professor, The Ohio State University) and Dr. Kay Russo (Veterinarian, RSM Consulting) gave updates. Dr. Russo gave a brief overview of the outbreak: in early March 2024, she received a call from dairy veterinarians who had discovered that some cattle had respiratory signs and fever, and that rumination had abruptly ceased. Furthermore, many animals had mastitis that was not associated with bacterial infection. After discovering that wild birds around the dairies were dying from H5N1, Dr. Russo suggested that the cattle also be tested for the virus, which subsequently came back as positive, and those were the first-ever cases of H5N1 diagnosed in dairy animals. In the field, clinical presentation and mortality percentages vary, with some herds having asymptomatic infection, whereas in other herds, up to 20% of the animals have clinical signs (26) for reasons that remain unknown. The virus is shed in the milk to very high levels (26) and is efficiently moving across the country with cattle movement. However, we still do not know how the virus is moving from farm to farm, or even cow to cow. Poultry populations are now at risk from strains circulating in bovine populations, as well as migratory bird populations. Unfortunately, it is still HPAI in poultry, and so as spillover into poultry continues, so too does the depopulation, and as a result, eggs are now expensive and hard to come by.

Dr. Bowman elaborated on the challenge studies his group has done in cows: intranasal challenge led to minimal replication in the respiratory tract and mammary gland and no evidence of transmission to co-housed chickens. In contrast, inter-mammary challenge was easily accomplished, and within a couple of days, the cows were euthanized for humane reasons. Other *in vivo* challenge studies have yielded similar results (34, 35). Furthermore, it has not been possible to reproduce transmission via contaminated milking equipment under experimental conditions, even though it is one of the leading hypotheses of how the virus is spreading on farms (36). Potentially, it is not a highly efficient route of transmission, and so with the small *n* numbers seen in experimental studies, it is hard to recapitulate consistently. Consequently, it is not possible to model potential interventions to put in place.

Dr. Russo explained current control strategies: in poultry, if an operation tests positive, the birds are depopulated as humanely as possible, as otherwise they would die from the infection. Next, a control zone is instituted around the operation within a 10 km radius, and everything in and out is tightly controlled. In cattle, there is not the same degree of mortality, so the approach to control it is different. However, as we have a poor understanding of how the virus is moving cow-to-cow, it is difficult to provide farm-level recommendations for producers. Compounding the issue, there is a lot of cattle movement in the US, creating a perfect situation for spreading the disease. Currently, there are efforts to quarantine farms, but there is still a lot of movement of animals and people on and off these farms, for example, non-lactating animals going to slaughter or dairy workers picking up shifts at poultry barns, etc. Dr. Russo emphasized that this is the first major infection event that the dairy industry has dealt with for decades, and biosecurity programs and protocols still need to be established and optimized. Vaccination of the animals needs to be heavily considered. However, the reason it is not yet implemented is in large part because of the implications on trade, and trade agreements would need renegotiating.

Regarding the situation in people, the majority of cases from dairy farms have been described as mild to moderate, with many patients presenting with conjunctivitis. However, there has been significant underreporting in farm workers. To date, those spillover events have been contained. At the time of writing, there have been 70 human cases of H5N1 in the US, including one death (41 from exposure to dairy herds, 24 from exposure to poultry, two from exposure to other animals, and three where the exposure source is unknown) (37). There has also been concern about people being infected with H5N1 from consuming milk. The majority of American homes have milk in the refrigerator, so protecting the milk supply is of paramount importance. Abnormal milk, or milk from sick animals, should not be going into the milk supply, so at present, that is waste milk, and, thankfully, several groups have demonstrated that pasteurization is effective in inactivating infectious virus in milk (38–41). However, despite these interventions, RNA from H5N1 is found in milk bought from the grocery store, and there is a risk of infection from drinking unpasteurized milk. One other way to control infection in people is personal protective equipment (PPE) for farm workers. However, they can make doing the job more challenging, which leads to low adoption. So, there needs to be a middle ground of what really is the most effective PPE and where to focus control efforts.

The panelists then took questions from the audience, with the first being whether cows could act like a “mixing vessel” for the emergence of reassortment strains. Dr. Bowman answered that this remains unknown, but as cattle have not typically been endemically infected with IAVs, there has not been anything to reassort with. However, as H5N1 has been detected in pigs (42), the veterinary public health community is concerned about more pig populations becoming infected with H5N1 and reassortment with endemic swine strains, so the connections between the dairy and swine industries should be further examined. Concerns were also raised from the audience regarding contamination of farm waste. Dr. Russo explained that dumping waste milk into manure lagoons has happened, and it is important to not spread that manure near poultry houses. Another question regarded whether cats were still becoming infected on dairy farms, and whether there has been any evidence of spread to rodents. Dr. Bowman replied that cats seem to be very susceptible and have been found with severe disease, including neurological signs (43, 44). Additionally, cats will sometimes disappear and may be found dead later in neighboring fields by farm workers picking vegetables, etc., so raising local awareness when the virus burden is high in the area is important. Dr. Russo expanded, describing how sometimes wildlife services will come in and euthanize different creatures surrounding an operation to test them, and they have found mice positive for H5N1, although how those contribute to the movement of the virus remains unclear, and the cats may be becoming infected by eating the mice or by drinking contaminated milk. One question on immunity referred to whether cattle were protected from reinfection after they recovered from a primary infection. Dr. Bowman stressed that there was no robust data on this yet. Dr. Russo expanded, describing how dairy farmers regularly replace approximately 30% of the herd on average yearly, and whether the continual replenishment of naïve animals into a herd helps perpetuate the virus still needs to be determined. The final question asked whether there have been any challenge studies done in cows to examine the transmission of the virus from cows to other mammalian hosts. Dr. Bowman replied that it was difficult to get cattle into containment to do those kinds of studies, but potentially they could be done in the future.

## CONCLUDING REMARKS

In summary, the symposium united experts across virus families, species, and career stages from academia, government, and industry, thus fostering cross-disciplinary discussion and networking, and the online discussion further connected veterinary clinicians with researchers. Strengthening these scientific networks benefits US agricultural research, which in turn improves the long-term sustainability of US food systems.
